# Information Dynamics of a Nonlinear Stochastic Nanopore System

**DOI:** 10.3390/e20040221

**Published:** 2018-03-23

**Authors:** Claire Gilpin, David Darmon, Zuzanna Siwy, Craig Martens

**Affiliations:** 1 Department of Physics and Astronomy, University of California-Irvine, Irvine, CA 92697-4575, USA; 2 Department of Military and Emergency Medicine, Uniformed Services University, Bethesda, MD 20814, USA; 3 Department of Chemistry, University of California-Irvine, Irvine, CA 92697-2025, USA

**Keywords:** entropy, local entropy rate, specific entropy rate, information dynamics, *k*-nearest neighbor estimation, nanopore

## Abstract

Nanopores have become a subject of interest in the scientific community due to their potential uses in nanometer-scale laboratory and research applications, including infectious disease diagnostics and DNA sequencing. Additionally, they display behavioral similarity to molecular and cellular scale physiological processes. Recent advances in information theory have made it possible to probe the information dynamics of nonlinear stochastic dynamical systems, such as autonomously fluctuating nanopore systems, which has enhanced our understanding of the physical systems they model. We present the results of local (LER) and specific entropy rate (SER) computations from a simulation study of an autonomously fluctuating nanopore system. We learn that both metrics show increases that correspond to fluctuations in the nanopore current, indicating fundamental changes in information generation surrounding these fluctuations.

## 1. Introduction

Over the previous three decades there has been an increasing interest in studying nanopore systems due to their broad applicability to the physical and biological sciences. Advances in nanometer-scale laboratory techniques have made it possible to create and isolate single nanopores, allowing detailed study of their properties. In addition to suggesting applicability as physiological models for molecular and cellular scale processes, and utility for DNA sequencing and rapid drug/microbial detection, their electronic behavior provides opportunities to study the information dynamics of nanoscale nonlinear oscillators [1,2,3,4,5,6,7,8,9,10,11,12,13,14,15,16,17].

Significant advances have been made in the development of tools to study information storage, processing, and transmission. Specifically, our understanding of Shannon entropy measures as metrics for information flow have progressed with notable momentum in the study of conditional (time-dependent) entropies, referred to as entropy rates in the literature. Local and specific entropy rates are among the most recent contributions in this area. These nonlinear measures of information dynamics can reveal interesting properties not accessible by classical linear methods such as autocorrelation and spectral analyses [18,19].

Local and specific entropy rates quantify complementary properties of a dynamical system. If we have a time series representing an observable, the local entropy rate (LER) represents the statistical surprise of seeing an already observed local future, given a specific past [20,21,22,23]. It also can be thought of as the rate of information generation at a given time point. The specific entropy rate (SER) represents the statistical uncertainty in an as yet unobserved future, given a specific past [24]. The LER, in isolation, is a retrospective measure, and the SER is a prospective measure, and each yields distinct information about the behavior of the dynamical system.

In [25], the authors use current–voltage data collected from a single, conical nanopore in contact with an ion solution to motivate a mechanistic model for the electronic behavior of the nanopore system. The pore is externally biased, but its fluctuations (openings and closings) are autonomous and result from dynamic formation and dissolution (or passage) of tiny inorganic precipitates [26,27]. They demonstrated that their mathematical model captures important properties of the observable from the nanopore system, including these fluctuations, which appear as rapid transitions between high and low conductance states in the time series. One reason this system is an ideal candidate for analysis via information dynamics is that its fluctuations may be informed by its previous states (transitions may depend on previous states, as they do in a Markov process).

The structure of this paper is as follows. In Section 2, we present the definitions and estimation procedures for local and specific entropy rates. We introduce the model of the nanopore in Section 3. We then investigate the properties of the nanopore system as viewed through the lens of information dynamics in Section 4. Finally, in Section 5, we conclude by considering other potential avenues of study for this and other nanoscale systems.

## 2. Definition and Estimation of Local and Specific Entropy Rates

### 2.1. Notation

Before beginning a discussion of the details of local and specific entropy rates, it is valuable to first establish standardized notation that will be used throughout this paper. We will be considering a stochastic process (current), ${\left\{X_{t}\right\}}_{t\in Z}$, in an autonomously fluctuating nanopore system. Realizations (or observed values) of this nanopore current will use lower case designation, $x_{1},x_{2},\ldots ,x_{t}$. A block of states will be denoted $X_{m}^{n}=\left(X_{m},X_{m+1},\ldots ,X_{n-1},X_{n}\right),$ where m<n.

Additionally, conditional densities are relevant to the discussion of local and specific entropy rates. Conditional densities will be expressed in the form $f_{Y|X}\left(a|b\right)$, which can be read as “the predictive density of *Y* conditioned on X=b”. For example, the predictive density of the future $X_{t}$, conditioned on a specific *p*-step past $x_{t-p}^{t-1}$ can be expressed as $f_{X_{t}|X_{t-p}^{t-1}}\left(x_{t}|x_{t-p}^{t-1}\right)$. We will assume conditional stationarity [28] of the process and thus denote the predictive density without time dependence as $f(x_{t}|x_{t-p}^{t-1})$. Note that this is a weaker assumption than strong stationarity which requires that joint distributions of any length are invariant under translation in time. Instead, we only assume that the distribution of the future, conditional on a sufficiently long past, is statistically invariant under translation in time.

### 2.2. Differential Entropy and Total Entropy Rate

In what follows, we assume familiarity with information theory at the level of [29]. Both local and specific entropy rates are born from differential entropy, and it is therefore important to introduce these quantities from the outset. Differential entropy can be thought of as the average uncertainty about the future if the model for a particular stochastic process is known, but the past is not. The definition of differential entropy for a random vector X is

(1) $$\begin{array}{cc} H[X] & =-E[\log f(X)]\\ & =-\int_{R^{n}}f(x)\log f(x)\;dx \end{array}$$

where f(x) represents the probability density of an *n*-dimensional distribution. If we know the past, we can condition the entropy, at any particular time point, on the past and obtain the following total entropy rate:

(2) $$\begin{array}{cc} H[X_{t}|X_{-\infty }^{t-1}] & =-E[\log f\left(X_{t}|X_{-\infty }^{t-1}\right)]\\ & =-\int_{R^{\infty }}\int_{R}f\left(x_{t},x_{-\infty }^{t-1}\right)\log f\left(x_{t}|x_{-\infty }^{t-1}\right)\;dx_{t}dx_{-\infty }^{t-1} \end{array}$$

where Equation (2) is defined as $\lim_{p\to \infty }H\left[X_{t}|X_{t-p}^{t-1}\right]$, if the limit exists. This quantity averages over all pasts and all futures to compute the uncertainty, but what if we want to know, at any given time point, our surprise given the realized past and future? We can answer this question with the LER.

### 2.3. Local Entropy Rate

The LER [20,21,22,23] is a time-dependent, locally-determined conditional entropy. The LER quantifies the surprise of observing a future $x_{t}$ given its preceding values. The LER is defined as the expectand (the expression internal to the expectation operator) of the total entropy rate evaluated at a particular past–future pair,

(3) $$H_{t}^{L}=H^{L}\left(x_{t}|x_{-\infty }^{t-1}\right)=-\log f\left(x_{t}|x_{-\infty }^{t-1}\right).$$

In practice, however, the density *f* is not known, and Equation (3) is not directly calculable. We must therefore find an estimator for LER through computation of an estimator for the predictive density. Moreover, because we always estimate *f* using finite data, it is not tractable to take the block of past values infinitely far into the past, and we therefore also require an appropriate model order *p*.

The first step in computing the estimator for LER is to determine the model order *p* for the predictive density $f(x_{t}|x_{t-p}^{t-1})$ used in its calculation. Conceptually, *p* quantifies the number of preceding state values that are necessary to determine the predictive density of $X_{t}$. In analyzing stochastic systems, the goal is to determine whether information contained in the past is useful to understanding the immediate future. If we do not look back far enough, we may miss useful information from the past that could help us determine the future. If we look too far back, we may inadvertently include information not relevant to determining the immediate future. As a concrete example, consider the case where the observable is well-modeled by a Markov process of some order *p*. In that case, after knowing the previous *p* values of the process, the future is independent of any values further in the past, so those values do not aid in the prediction of the process. However, they do increase the burden of the associated estimation problem, through the curse of dimensionality [30]. That is, geometrically more data is necessary to achieve the same level of precision in the estimate of the density. In the case where the process is not Markovian, a similar argument applies, except with the added consideration for balancing between the contribution of including more of the past and its increasing burden to estimation process. We seek the model order that results in a minimum uncertainty. Using the information theoretic criterion from [31], the model order is chosen to minimize the negative log predictive likelihood (NLPL)

(4) $$\mathrm{NLPL}\left(p\right)=-\frac{1}{N-p}\sum_{i=p+1}^{N}\log{\hat{f}}_{-i}\left(X_{i}|X_{i-p}^{i-1}\right)$$

where *N* is the number of points in the time series, and ${\hat{f}}_{-i}$ is an estimator of the predictive density estimated holding out the block $X_{i-p}^{i}$. We use a kernel-nearest neighbor estimator for *f*, which performs kernel density estimation over the set of nearest neighbors in the future space [32,33]. The estimator takes the form

(5) $${\hat{f}}_{-i}\left(X_{i}|X_{i-p}^{i-1}\right)=\frac{1}{J}\sum_{m\in N_{J}\left(X_{i-p}^{i-1}\right)}K_{h}\left(X_{i}-X_{m}\right)$$

where $K_{h}$ is a Gaussian kernel, *J* is the number of nearest neighbors to $X_{i-p}^{i-1}$, $N_{J}\left(X_{i-p}^{i-1}\right)$ is the index set of the *J*-nearest neighbors, and *h* is a bandwidth for the density estimator over futures. Equation (4) over *h*, *J*, and *p* is optimized using the constrained Nelder–Mead method from the NLopt library, where *h* is constrained to (0,∞) and *J* is constrained to $\left\{1,\ldots ,J_{max}\right\}$, where $J_{max}\ll N$ [34].

Once the optimal model order is determined, it is used to compute the estimator for the LER at time $t_{i}$:

(6) $$\begin{array}{c} {\hat{H}}_{t_{i}}^{L}=-\log\left\{\frac{\hat{f}\left(X_{i},X_{i-p}^{i-1}\right)}{\hat{f}\left(X_{i-p}^{i-1}\right)}\right\} \end{array}$$

where the density estimators are computed using a *k*-th-nearest neighbor method [35]. A free-standing LER estimator (an estimator of the LER that will not be subsequently used to compute an estimator of the SER) must be computed using a value of *k* that scales with a power of *N* to ensure consistency in the estimation of the densities [35]. In this work, $k\left(N\right)=\left\lfloor \sqrt{N}\right\rfloor$ was used. As we will see in the next section, the estimator of LER is also used in the estimator of the SER. In that case, the LER estimator may be more efficiently computed using a smaller *k* due to the impact of averaging on the bias–variance trade-off. In that case, we use k=4 in this work.

### 2.4. Specific Entropy Rate

The SER is a measure of the predictive uncertainty in the future conditional on a specific past. Unlike the LER, which is retrospective, the SER is completely predictive because it averages over futures to compute the uncertainty. The SER is an average of LER values conditioned on a specific past. It takes the following form [24,36]:

(7) $$\begin{array}{cc} H_{t_{i}}^{S} & =H^{S}\left[X_{i}|X_{i-p}^{i-1}=x_{i-p}^{i-1}\right]\\ & =-E[\log f\left(X_{i}|X_{i-p}^{i-1}\right)|X_{i-p}^{i-1}=x_{i-p}^{i-1}]\\ & =-\int_{R}f\left(x_{i}|x_{i-p}^{i-1}\right)\log f\left(x_{i}|x_{i-p}^{i-1}\right)\;dx_{t}. \end{array}$$

Similarly, for practical purposes, an estimator for the SER is computed via averaging of the estimator for the LER. Once the LER estimator values have been computed, $k^{*}$ of them are averaged at each time point to obtain the estimator for the SER at time $t_{i}$:

(8) $${\hat{H}}_{t_{i}}^{S}=\frac{1}{k^{*}}\sum_{j\in N_{k^{*}}\left(X_{i-p}^{i-1}\right)}{\hat{H}}^{L}\left(X_{j}|X_{j-p}^{j-1}\right)$$

where again $N_{k^{*}}\left(X_{i-p}^{i-1}\right)$ is the index set of the $k^{*}$-nearest neighbors of $X_{i-p}^{i-1}$. It is important to note that $k^{*}$ is distinct from *k* in the previous discussion of LER. $k^{*}$ is set at $\left\lfloor \sqrt{N}\right\rfloor$ in this work to ensure consistency of the estimator [35,37,38,39].

### 2.5. Specific Information Dynamics with Python

The code for the determination of model order and the estimation of local, specific, and total entropy rates is available in the Specific Information Dynamics with Python (sidpy) package, hosted on GitHub [40].

## 3. Model System

**Figure 1:**
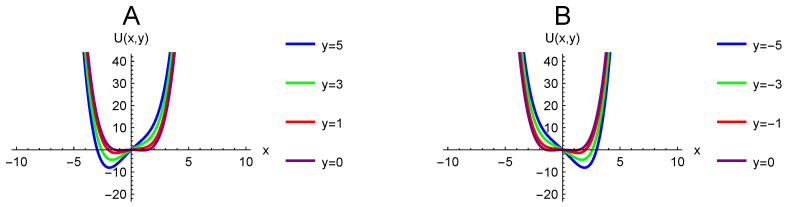
Potential at fixed positive values of *y* (**A**) and fixed negative values of *y* (**B**). The y=0 configuration is shown on both plots. As *y* becomes more positive, it causes the potential to skew towards a transition to negative *x*. As *y* becomes more negative, it causes the potential to skew towards a transition to positive *x*. Physically, positive values of *x* in this graph correspond to positive current values.

We consider the information dynamics of a nanopore current oscillator, empirically observed and modeled in [25,26,27,41]. A single nanopore current oscillator, which is observed in a single state *x* as a function of time *t*, can be modeled as a coupled nonlinear oscillator with bistable potential U(x,y) forced by dynamical noise:

(9) $$\begin{array}{cc} dX_{t} & =-\frac{1}{\gamma_{x}}\frac{\partial U}{\partial x}\left(X_{t},Y_{t}\right)\;dt+\sigma_{x}\;dW_{x}\\ dY_{t} & =\frac{1}{\gamma_{y}}\left[k_{+}\Theta \left(X_{t}\right)-k_{-}\Theta \left(-X_{t}\right)\right]dt+\sigma_{y}dW_{y} \end{array}$$

where $X_{t}$ is the nanopore current, $Y_{t}$ is an unobserved state variable that controls the opening and closing behavior of the nanopore, γ is a coefficient of friction, Θ is a Heaviside function with amplitude determined by rate constants $k_{+}$ and $k_{-}$, and $W_{x}$ and $W_{y}$ are standard Brownian motions representing other unaccounted for inputs to the system. The double-well potential U(x,y) is taken to be

(10) $$U\left(x,y\right)=\frac{1}{4}ax^{4}-\frac{1}{2}b\left(V\right)x^{2}+cxy$$

where b(V) is the voltage-dependent parameter that determines the barrier height:

(11) $$b\left(V\right)=b_{0}\left(\frac{V-V_{c}}{V_{c}}\right)$$

with $V_{c}$ as the critical voltage. The behavior of the potential as it relates to both *X* and *Y* is critical to understanding the behavior of the nanopore current and should be examined in more detail. When *X* takes positive values, *Y* will be a random walk with drift $\frac{k_{+}}{\gamma_{y}}$ plus dynamical noise. As *Y* drifts to more positive values, the potential tips towards the negative well, making it likely that a transition will occur from a positive to a negative current. This effect can be seen in Figure 1A below. Conversely, when the nanopore current *X* takes negative values, *Y* will be a random walk with drift $\frac{-k_{-}}{\gamma_{y}}$ plus dynamical noise. Eventually, *Y* drifts negative to a point of tipping the potential towards the positive well, making it likely that a transition will occur from a negative current to a positive current. This effect can be seen in Figure 1B below. Between fluctuations, *Y* will pass through Y=0.

The structure of the potential term as it relates to *X* and *Y* acts to ensure that the system undergoes transitions frequently and never becomes stuck indefinitely in one of the wells. Similar behavior of the nanopore current should therefore be expected across realizations of data simulated using this model, despite expected differences in the profiles of individual transitions due to the dynamical noise.

For the simulation, the external bias voltage *V* is held constant. Values used in this work are a=b=c=1, $\gamma_{x}=1$, $\gamma_{y}=100$, $k_{+}=1$, $k_{-}=5$, and $\sigma_{x}=\sigma_{y}=0.1$ [25]. Realizations from Equation (9) were simulated using the SRI2 stochastic integrator from [42] with a time step of 0.25. The LER and SER were computed for each realization after downsampling to a time step of 0.5, resulting in a total of 40,000 time points.

## 4. Results

**Figure 2:**
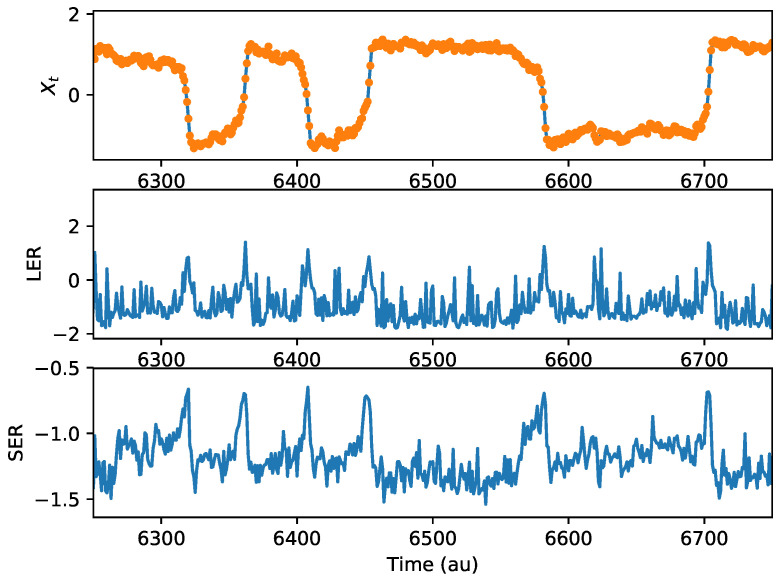
**Top**: the nanopore current, with each orange dot representing a measurement. **Middle**: the estimate of the local entropy rate (LER) of the nanopore system as a function of time. **Bottom**: the estimate of the specific entropy rate (SER) of the nanopore system as a function of time. This is a representative excerpt from a 40,000 point time series containing on the order of 100 transitions.

Five separate realizations of the stochastic differential Equation (9) were computed. Figure 2 shows the nanopore current, the LER, and the SER for a group of transitions in the nanopore system (pore open/close events). All three panels are aligned in time. The uppermost panel represents the nanopore current as a function of time. Each orange point is one measurement. Open/close (transition) events can be seen in the rapid switching of the nanopore current from positive to negative values or vice versa. The middle panel is the estimated free-standing LER, computed using Equation (6). We can see that there are peaks in the free-standing LER aligned with the transition events of the nanopore. This indicates that information is generated by these events, and there was some surprise associated with their occurrence. The bottom panel is the SER estimate computed via Equation (8). We see that the SER also increases around the transition events in the nanopore, indicating an increase in uncertainty about future states near the transitions. These results are a subset of results from a single realization, but are representative of the behavior seen in these information measures during transition events across all five realizations, as is expected for this model.

The peaks in the LER and SER corresponding to transitions should be considered in the context of the model. It will be easiest to make a transition between positive and negative currents when the slope of the potential is greater (i.e., when the magnitude of *y* is further from zero). Additionally, when the slope of the potential is large, any small kick from the dynamical noise could lead to a wider array of possible futures (noise amplification). There will accordingly be a greater uncertainty in an unseen future (higher SER) during the transition events. There will also be an elevated LER in these regions because, of the many possible outcomes for current values, when the potential slope is large, individual outcomes may occur only rarely. This will translate to a relatively high surprise.

It should also be noted that there are substantial peaks in the LER that are not always associated with transitions. The LER metric is sensitive to viewing any atypical future. In the relatively flatter (low variation in nanopore current) regions between transitions, any variation above the noise level from the anticipated trajectory may result in high surprise, even though a transition may not occur. This is particularly prominent about 6430 au and 6620 au in Figure 2. If the future is unseen, as in the SER, in these relatively flatter regions there will be low uncertainty about the future. In other words, variation above the noise level is not expected. This is why similar peaks not associated with transitions are rarely seen in the SER.

**Figure 3:**
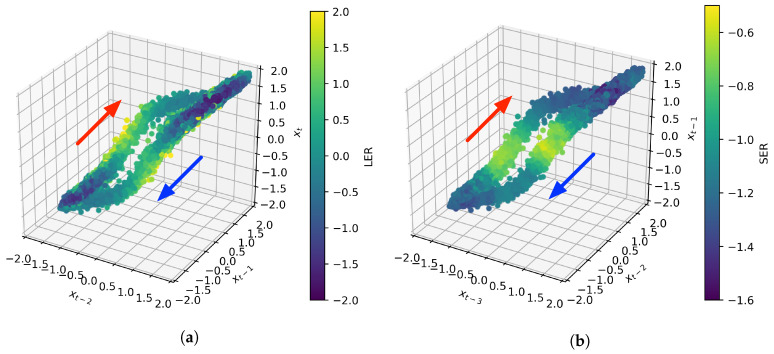
A projection of the reconstructed state space for the nanopore system shaded by the estimates of the LER (**left**) and SER (**right**) associated with the overall state. The plots reveal a clear trajectory in the reconstructed state space, and the arrows indicate the direction along the transitions between open and closed states. Along this trajectory, regions of relatively low surprise (LER) and low uncertainty (SER) occur when the system is in an open/closed state. Conversely, in the central regions, corresponding to transitions, we see increases in both the LER and SER. Anti-symmetry is noted in the onset of increase in SER, which shows that uncertainty is highest at the beginning of a transition and decreases as the transition proceeds to completion. (**a**) LER; (**b**) SER.

To further investigate transitions, we consider how the LER and SER vary as a function of the reconstructed state space of the nanopore system. Figure 3 shows a 3D projection of the p=4 reconstructed state space, where each point is shaded by the LER (left) and SER (right). We use the projection $\left(X_{t-2},X_{t-1},X_{t}\right)$ for the LER and $\left(X_{t-3},X_{t-2},X_{t-1}\right)$ for the SER. The arrows indicate the direction of the transitions with respect to time. We know that, if the nanopore is in a closed state, it is likely to remain closed and that, if it is in an open state, it is likely to remain open. We thus see relatively low surprise (a low LER) and relatively low uncertainty (a low SER) under those conditions, corresponding to the points in the bottom left and top right of the reconstructed state space. When a transition event is occurring, corresponding to the points along the central “tubes”, we are relatively more surprised (a higher LER) and relatively more uncertain about the immediate future (a higher SER). It should also be noted that, if all transitions in this system were identical, the reconstructed state-space trajectory would not show spread about the average path (i.e., the “fuzziness” is due to differences in the profile of the transitions). Although the LER and SER have similar regions of relatively high/low values in their corresponding measures, it is important not to conflate them. The LER measures information generated by seeing the future, and it should not surprise us to see a high degree of symmetry in the LER plot, with maximal information generated for more atypical transitions (on the outsides of the transition “tubes”). The SER, by contrast, measures uncertainty in the future, given a known past, and we might expect high uncertainty in the region of all transitions. It is additionally noteworthy that there is some anti-symmetry between the two transition tubes in the SER plot with respect to the location of the onset of elevation in the SER. This, together with the arrows indicating the direction of the trajectory with time, shows that uncertainty is highest at the beginning of a transition. Uncertainty decreases as the transition proceeds to completion. This is not apparent from examination of the time series.

**Figure 4:**
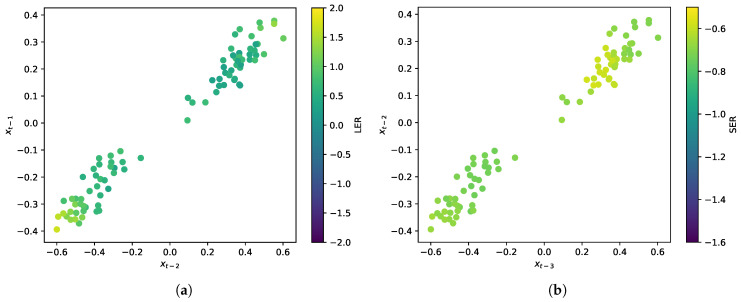
A 2D cross section of the reconstructed state space constructed from points within ϵ=±0.05 of the $x_{t}=0$ (**left**) and $x_{t-1}=0$ (**right**) planes for each plot, respectively. These plots show that information is generated most heavily around atypical transition events (the highest LER visible on the periphery of the transition tubes in the LER plot), and there is relatively uniform, high uncertainty for all transitions in the SER plot. (**a**) LER; (**b**) SER.

It may also be helpful to look directly at the transition region in both the LER and SER schemes, i.e., to look inside the trajectory in the transition regions. To do so, we take a cross section of the plots in Figure 2 at $x_{t}=0$ and $x_{t-1}=0$, respectively, and include points that fall within a tolerance of ϵ=±0.05. This cross section is shown in Figure 4. We can see that the LER is highest in the regions corresponding to less typical transitions (i.e., on the outside of the tubes), as previously mentioned. Additionally, as expected, all transitions in the SER scheme are associated with a similarly elevated SER.

## 5. Discussion

At this point it is worth considering what we have learned from calculating both the LER and SER that we could not have otherwise learned from visually examining the time series. It would seem obvious that state transitions in this nanopore system (i.e., pore open or closed) can be observed through such visual examination, but changes in the information dynamics brought about by those transitions cannot be gleaned through such observation. The fact that both the LER and the SER vary with transition behavior suggests that the transitions observed in the time series reflect deeper changes in the information dynamics of the governing system. This is in contrast with systems where transitions in the time series occur in the absence of changes in information status, such as fully deterministic systems or systems where all transitions are alike.

We are able to better understand the complementary value of the LER and SER through visualization of their values in the reconstructed state space. We see that the maximum uncertainty, associated with the maximum SER, occurs when the system is undergoing a transition between the open and closed pore configurations. The uncertainty increased at the start of transitions and falls as they proceed to completion. We also see that surprise, represented by the LER, is concentrated in the transition region, but also has secondary peaks occurring in regions with less typical futures.

Information metrics, such as the LER and SER, provide us with new insights into the underlying dynamics of nanopore systems. This may prove useful in the future to understanding their autonomous fluctuations and the behavior of other systems modeled by similar mathematics. A natural direction for a future study would be to extend these analytical techniques to empirical data from a nanopore system or to empirical data from biological systems, such as gated ion channels. Studying these nanoscale systems using information dynamics is, to the authors’ knowledge, a novel activity, and may reveal interesting properties of these systems not previously accessible. With this new exploration comes the possibility that these techniques will help illuminate nanoscale biological processes.

## Abbreviations

The following abbreviations are used in this manuscript:

LER	Local entropy rate
SER	Specific entropy rate
NLPL	Negative log predictive likelihood
